# Risk factor-based analysis of community-acquired pneumonia, healthcare-associated pneumonia and hospital-acquired pneumonia: Microbiological distribution, antibiotic resistance, and clinical outcomes

**DOI:** 10.1371/journal.pone.0270261

**Published:** 2022-06-29

**Authors:** Hakjun Hyun, Joon Young Song, Jin Gu Yoon, Hye Seong, Ji Yun Noh, Hee Jin Cheong, Woo Joo Kim

**Affiliations:** Division of Infectious Diseases, Department of Internal Medicine, Korea University College of Medicine, Seoul, Republic of Korea; Shandong Public Health Clinical Center: Shandong Provincial Chest Hospital, CHINA

## Abstract

**Background:**

Healthcare-associated pneumonia (HCAP) lies in the intersection of community-acquired pneumonia (CAP) and hospital-acquired pneumonia (HAP). Although HCAP is excluded from the revised HAP guideline, reassessment for HCAP is needed considering its heterogeneous characteristics.

**Methods:**

The microbiological distribution, antibiotic resistance, and clinical outcomes in CAP, HCAP, and HAP were studied retrospectively. The susceptibility to standard CAP regimens (β-lactams plus macrolide or fluoroquinolone monotherapy) and rates of methicillin-resistant *Staphylococcus aureus* (MRSA) and *Pseudomonas aeruginosa* (*P*. *aeruginosa*) infections were evaluated in the CAP group and HCAP subgroups.

**Results:**

In total, 933 cases were included (CAP, n = 557; HCAP, n = 264; HAP, n = 112). In the CAP and HCAP cases, *Streptococcus pneumoniae* (7.4% vs. 5.7%) and *P*. *aeruginosa* (9.2% vs. 18.6%) were the most common gram-positive and gram-negative pathogens. *Staphylococcus aureus* (methicillin-resistant, 2.7%; methicillin-susceptible, 2.4%) and carbapenem-resistant *Acinetobacter baumannii* (20.5%) were the most common Gram-positive and Gram-negative pathogens in the HAP group, respectively. Higher susceptibility to levofloxacin was observed in CAP and HCAP isolates than that to β-lactam agents. However, levofloxacin non-susceptibility was significantly higher in long-term care facility (LTCF)-onset HCAP compared to community-onset HCAP (43.6% vs. 22.7%, *P* = 0.014).

**Conclusion:**

HCAP showed higher rates of *P*. *aeruginosa* and MRSA infections than CAP. Empirical antipseudomonal therapy should be considered in the treatment of HCAP. Prior isolation of *P*. *aeruginosa* was the most important risk factor for *P*. *aeruginosa* infection.

## Introduction

Pneumonia is the third leading cause of death worldwide. In particular, the morbidity and mortality of pneumonia are much higher among the elderly aged 65 years or older. Therefore, it is an important issue because of aging population in many countries, including South Korea [1].

Pneumonia is classified into community-acquired pneumonia (CAP) and nosocomial pneumonia. Because of the possibility of multidrug-resistant (MDR) pathogens, the 2005 American Thoracic Society/Infectious Diseases Society of America (ATS/IDSA) guidelines for nosocomial pneumonia recommended broad-spectrum empirical antibiotics for the three types of pneumonia: hospital-acquired pneumonia (HAP), ventilator-associated pneumonia, and healthcare-associated pneumonia (HCAP). The risk factors for HCAP were defined as patients who were residents in a nursing facility, those who were hospitalized for more than 2 days within the past 90 days, and those who had recently received intravenous antibiotics, chemotherapy, home wound care, and hemodialysis within the past 30 days [2]. However, as studies conducted since 2005 reported that there was no significant difference in the distribution of causative agents between HCAP and CAP, the ATS/IDSA guidelines in 2016 no longer recommended broad-spectrum antibiotics for HCAP [3, 4].

Despite the change in the guidelines, the HCAP criteria of ATS/IDSA showed high sensitivity (89%) and high negative predictive value (96%) in predicting MDR bacterial infection and colonization [5]. These criteria can be easily applied to clinical practice for the risk assessment of MDR bacterial infections. However, there are still many reports showing that the distribution of pathogens causing HCAP is different from that of CAP [6–8]. With increasing medical service use, along with an aging population, MDR pathogens are more likely to be prevalent. Therefore, a detailed classification and reassessment are necessary for HCAP. HCAP includes a variety of patient groups and has heterogeneous characteristics. Therefore, the microbiological characteristics of each pneumonia group (CAP, HCAP, and HAP) and risk factors for antibiotic-resistant infection would be helpful in predicting antibiotic-resistant pathogens and clinical outcomes.

This study aimed to compare the microbiological distribution, antibiotic resistance, and clinical outcomes in cases of CAP, HCAP, and HAP. In addition, we evaluated the prevalence of MDR pathogens among the HCAP subgroups and the risk factors for antibiotic-resistant bacterial infection in the cases of CAP and HCAP.

## Methods

### Study design

This was a retrospective single-center study of patients with pneumonia hospitalized at Korea University Guro Hospital (a 1,075 bed [annually 41,000 admissions], university-affiliated, urban, tertiary referral hospital in Seoul, South Korea) from July 1, 2018 to June 30, 2019. Adults aged >18 years were included. The cases were extracted through a hospital-based database of pneumonic patients and through a list of patients with ICD-10 codes (J12-J18). All cases were reviewed by two experienced infectious disease doctors. We excluded patients who were transferred to other hospitals and refused life-sustaining treatment. In cases of recurrent pneumonia during the study period, the first event was included in the analysis.

The objectives of this study were as follows: (1) to compare the microbiological distribution of causative agents and clinical outcomes among cases of CAP, HCAP, and HAP; (2) to compare the rates of antibiotic-resistant infection between the CAP group and the HCAP subgroups; (3) to compare the susceptibility of CAP and HCAP isolates to standard CAP regimens (4) to analyze the risk factors for antibiotic-resistant bacterial infection. We evaluated the risk factors for *Pseudomonas aeruginosa (P*. *aeruginosa)* infection, which is important when selecting the empirical antibiotics for pneumonia.

The study was approved by the ethics committee of Korea University Guro Hospital (IRB No. 2020GR0398), which waived the need for informed consent, and was conducted in accordance with the Declaration of Helsinki and Good Clinical Practice. All data were fully anonymized before we accessed them.

### Data collection

Data were collected by reviewing the medical records and laboratory results of the study subjects. Two experienced researchers reviewed the medical records. The collected data included demographic information, medical comorbidities, antibiotic resistance-related risk factors (prior intravenous antibiotic use, hospitalization, long-term care facility (LTCF) residence, hemodialysis, chemotherapy, and prior *P*. *aeruginosa* and methicillin-resistant *Staphylococcus aureus* (MRSA) isolation from respiratory samples within a year), medical device use (tracheostomy and tube feeding), pneumonia severity (presence of shock and use of a mechanical ventilator), empirical antibiotic regimen, antibiotic treatment duration, clinical outcomes (30-day mortality, hospitalization duration, and rehospitalization within a year), and pneumonia-related complications (lung abscess, empyema, acute rhabdomyolysis, cardiovascular events, and acute kidney injury). With regards to the prior antibiotic exposure in HAP cases, antibiotic use within 90 days from the onset of HAP was collected. The Charlson comorbidity index, CURB-65 score, and pneumonia severity index (PSI) were calculated using clinical epidemiological data collected from the patients.

To identify the causative agents of pneumonia, microbiological data within 3 days after the diagnosis of pneumonia were collected: sputum culture, blood culture, respiratory viral polymerase chain reaction (PCR, Anyplex® Seegene), pneumonia bacterial PCR (Seeplex® Seegene), urinary antigen tests (*Streptococcus pneumoniae* and *Legionella pneumophila* serogroup 1), and serologic tests (*Mycoplasma*, *Legionella*, and *Chlamydophila* spp.). As for the sputum culture, it was considered as the causative agents when those were consistent with the results of Gram stain. In South Korea, Health Insurance Review and Assessment Service (HIRA) introduced the program for the quality assessment of pneumonia care in 2015, so blood culture, sputum culture, and sputum Gram stain were routinely performed for pneumonic patients within 24 hours on admission. As for the pneumonia bacterial PCR, we only regarded atypical pathogens (*Mycoplasma*, *Legionella*, and *Chlamydophila*) as the causative agents of pneumonia.

As for the species identification and antimicrobial susceptibility tests, VITEK II automated system (bioMérieux Inc.) was used, utilizing a standard identification card and the modified broth microdilution method. Susceptibility tests were interpreted according to Clinical and Laboratory Standards Institute (CLSI) guidelines [9]. Particularly, the susceptibility of the causative agents for ceftriaxone, ampicillin-sulbactam, and levofloxacin was investigated.

### Case definition

Pneumonia was defined according to the 2005 guidelines of the ATS/IDSA, showing newly visible lung infiltrates on chest radiographs and accompanying symptoms (cough, exudative sputum, fever, and dyspnea) or leukocytosis on blood tests [2]. In addition, pneumonia was classified into CAP (excluding HCAP), HCAP, and HAP. Pneumonia that occurred more than 48 h after admission was defined as HAP. Ventilator-associated pneumonia (VAP) was included in HAP. The time point of HAP onset was identified based on medical records and chest imaging: sputum Gram stain, culture and antibiotic treatment. Pneumonia that occurred within 48 h after hospital admission and having any of the following risk factors was defined as HCAP: prior hospitalization for more than 2 days within the past 90 days, prior intravenous antibiotic use within the past 90 days, residence at LTCF, hemodialysis, chemotherapy, and wound treatment within the past 30 days. Pneumonia that occurred within 48 h after hospitalization in the absence of the above risk factors was defined as CAP. HCAP was further classified into four subgroups according to the history of LTCF residence, prior hospitalization, and prior antibiotic use. Subgroup 1 included residents of LTCF without prior hospitalization and antibiotic use within the past 90 days. Subgroup 2 included LTCF residents with prior hospitalization or antibiotic use within 90 days. Subgroup 3 included non-LTCF residents with prior hospitalization or antibiotic use within the past 90 days. Subgroup 4 included non-LTCF residents who did not have prior hospitalization and antibiotic use within the past 90 days but received chemotherapy or hemodialysis within the past 30 days. According to this classification, subgroup 1 and 2 were defined as LTCF-onset HCAP and subgroup 3 and 4 were defined as community-onset HCAP.

The causative microbial agents were classified according to antibiotic susceptibility to the standard empirical regimen recommended in the 2019 ATS/IDSA guidelines. The standard empirical antibiotic regimens for CAP are β-lactams (ampicillin-sulbactam, cefotaxime, ceftriaxone, and ceftaroline) plus macrolides (azithromycin and clarithromycin), or fluoroquinolone (levofloxacin and moxifloxacin) monotherapy [10]. According to this classification, microbial agents susceptible to the standard CAP regimen include *S*. *pneumoniae*, methicillin-sensitive *Staphylococcus aureus* (MSSA), *Enterobacter* species (spp.), *Enterococcus* spp., *Haemophilus influenzae*, extended-spectrum beta lactamase (ESBL)-nonproducing Enterobacterales (*Klebsiella pneumoniae* and *Escherichia coli*), *Moraxella catarrhalis*, *Proteus mirabilis*, *Serratia marcescens*, and atypical organisms (*Chlamydophila pneumoniae*, *Mycoplasma pneumoniae*, and *Legionella pneumophilia*). Organisms not susceptible to the standard CAP regimen include *P*. *aeruginosa*, MRSA, ESBL-producing Enterobacterales, carbapenem-resistant *Acinetobacter baumannii* (CRAB), and vancomycin-resistant *Enterococcus* spp. *P*. *aeruginosa* was considered not susceptible to the standard CAP regimen, even though it was susceptible to fluoroquinolones; *P*. *aeruginosa* is not susceptible to β-lactams (ampicillin-sulbactam, cefotaxime, ceftriaxone, and ceftaroline) plus macrolides. When two or more causative microbial agents were identified, it was defined as polymicrobial infection. The cases of polymicrobial, viral, and fungal infections were excluded from the classification of susceptibility to the standard CAP regimen.

With regard to pneumonia-related complications, acute kidney injury was defined by the 2012 Kidney Disease Improving Global Guideline as an elevation of serum creatinine concentration more than 1.5 times the patient’s baseline [11]. Lung abscess and empyema were defined as patients with corresponding findings on chest radiography or chest computed tomography taken during hospitalization. Shock was defined as a decrease in systolic blood pressure below 90 mmHg or the use of vasopressors or inotropic agents. Rhabdomyolysis was defined as an elevation of serum creatine phosphokinase more than five times the normal reference value.

### Statistical analysis

Statistical analysis was conducted using the Statistical Package for the Social Sciences version 20 (SPSS Inc., Chicago, IL, USA). The chi-square test or Fisher’s exact test was used for categorical variables, while Student’s t-test or one-way analysis of variance (ANOVA) test was used for continuous variables to compare the differences between the groups. After one-way ANOVA, post-hoc analysis was performed using Tukey’s method. For the comparison of the CAP group and the HCAP subgroups, CAP was used as a reference group and compared with the four subgroups of HCAP. Binary logistic regression analyses were used to identify the risk factors for *P*. *aeruginosa* infection in CAP and HCAP cases, as measured by the odds ratio (OR) with 95% confidence intervals (CIs). Statistical significance was set at *P*<0.05.

## Results

### Baseline characteristics

**Fig 1:**
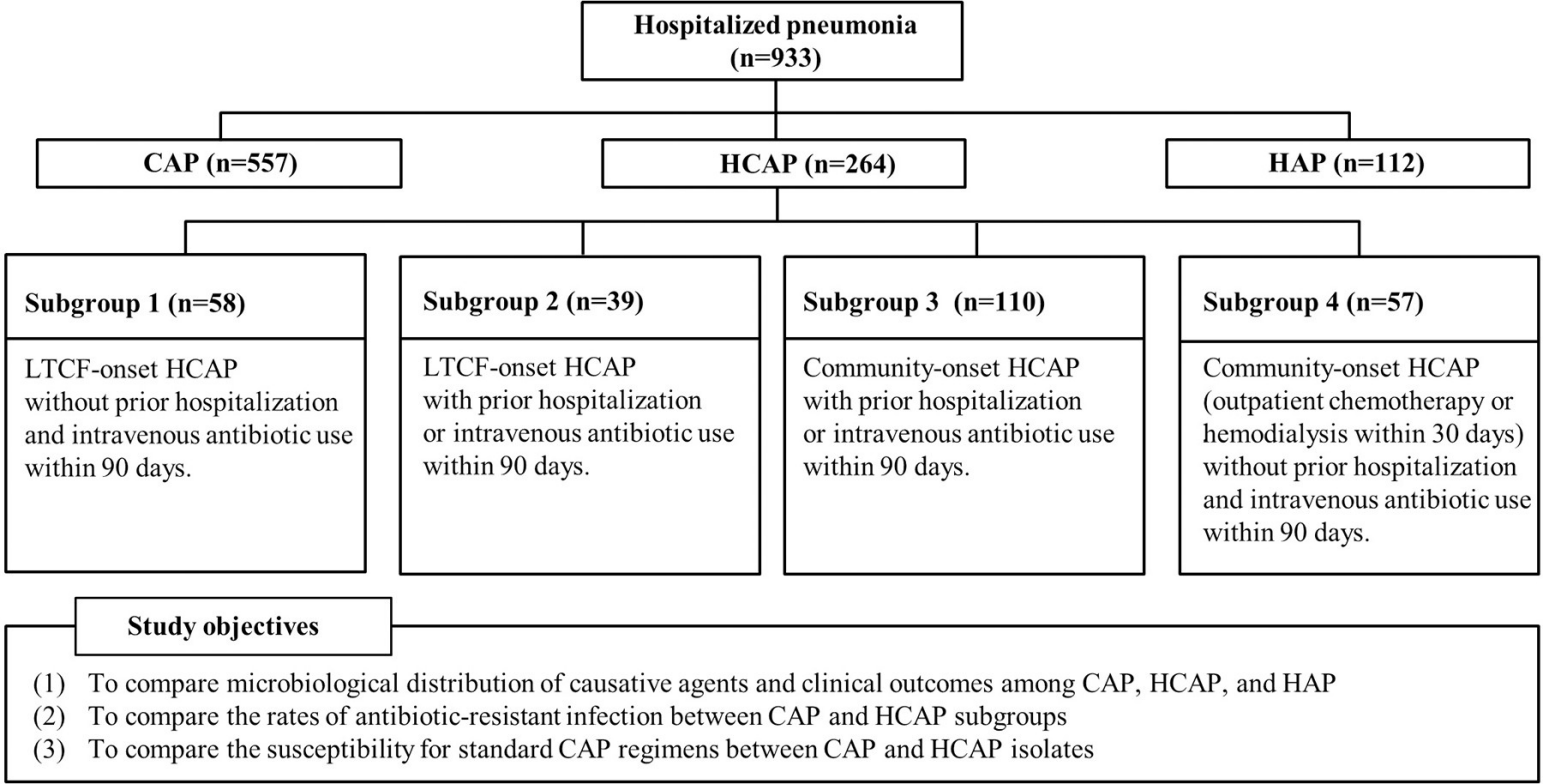
Study diagram.

A total of 933 hospitalized patients with pneumonia were included in this study (Fig 1). The mean age of the patients was 70.4 years, and males accounted for 64% (360 cases of CAP, 157 of HCAP, and 80 of HAP). Of the 933 cases, 557 presented with CAP, 264 with HCAP, and 112 with HAP. Patients with HCAP and HAP had more medical comorbidities than those with CAP (Table 1). The Charlson comorbidity index was highest in the HCAP cases, followed by the HAP and CAP (5.3±2.6 vs. 4.8±2.7, and 4.0±2.5, respectively; *P*<0.001). The HCAP cases were more likely to have antibiotic resistance-related risk factors than the HAP cases, including prior intravenous antibiotic use within the past 90 days (38.6% vs. 27.7%, *P* = 0.042), prior hospitalization within the past 90 days (46.2% vs. 23.2%, *P*<0.001), LTCF residence (36.7% vs. 13.4%, *P*<0.001), chemotherapy (20.5% vs. 6.3%, *P* = 0.001), and hemodialysis (13.3% vs. 0.9%, *P*<0.001).

**Table 1:** Baseline characteristics of hospitalized patients with pneumonia.

	CAP (n = 557)	HCAP (n = 264)	HAP (n = 112)	*p-*value
Demographics				
Male sex, No. (%)	360 (64.6)	157 (59.5)	80 (71.4)	0.077
Age, year (mean±SD)	70.0±15.4	71.8±12.8	69.0±15.5	0.161
BMI	22.4±4.0	21.8±4.3	22.5±4.6	0.229
Medical comorbidities, No. (%)				
Chronic heart diseases	30 (5.4)^a^	26 (9.8)^b^	15 (13.4)^b^	0.004
Neurovascular diseases	81 (14.5)^a^	83 (31.4)^b^	39 (34.8)^b^	<0.001
Chronic renal diseases	41 (7.4)^a^	50 (18.9)^b^	15 (13.4)^b^	<0.001
Diabetes	166 (29.8)	92 (34.8)	38 (33.9)	0.303
COPD	85 (15.3)^a^	30 (11.4)^a^	4 (3.6)^b^	0.002
Asthma	26 (4.7)	14 (5.3)	2 (1.8)	0.309
Structural lung diseases	53 (9.5)^a^	13 (4.9)^b^	3 (2.7)^b^	0.008
Liver cirrhosis	17 (3.1)	6 (2.3)	2 (1.8)	0.668
Hematologic malignancy	7 (1.3)^a^	5 (1.9)^ab^	6 (5.4)^b^	0.016
Solid cancer	109 (19.6)^a^	91 (34.5)^b^	32 (28.6)^b^	<0.001
Rheumatologic diseases	13 (2.3)	4 (1.5)	2 (1.8)	0.725
Immunosuppressant use	41 (7.4)^a^	16 (6.1)^a^	0 (0.0)^b^	0.012
Solid organ transplantation	3 (0.5)	1 (0.4)	1 (0.9)	0.823
HIV	2 (0.4)	0 (0)	0 (0)	0.508
Splenectomy state	1 (0.2)	0 (0)	0 (0)	0.713
Charlson Comorbidity Index	4.0±2.5^a^	5.3±2.6^b^	4.8±2.7^b^	<0.001
Medical device use, No. (%)				
Tracheostomy*	3 (0.5)^a^	11 (4.2)^b^	3 (2.7)^ab^	0.001
Tube feeding	5 (0.9)^a^	24 (9.1)^b^	3 (2.7)^a^	<0.001
Antibiotic resistance related risk factor, No. (%)				
Prior IV antibiotic use within 90 days	0 (0)^a^	102 (38.6)^b^	31 (27.7)^c^	<0.001
Long-term care facility	0 (0)^a^	97 (36.7)^b^	15 (13.4)^c^	<0.001
Prior hospitalization within 90 days	0 (0)^a^	122 (46.2)^b^	26 (23.2)^c^	<0.001
Chemotherapy	0 (0)^a^	54 (20.5)^b^	7 (6.2)^c^	<0.001
Hemodialysis	0 (0)^a^	35 (13.3)^b^	1 (0.9)^c^	<0.001
Prior MRSA isolation	12 (2.0)	14 (5.1)	1 (0.9)	0.017
Prior *P*. *aeruginosa* isolation	26 (4.7)	18 (7.1)	3 (2.8)	0.201
Pneumonia severity				
CURB65, median (IQR)	2 (1–2)^a^	2 (1–3)^b^	2 (1–3)^b^	<0.001
Pneumonia severity index, mean±SD	106.8±36.1^a^	123.5±39.9^b^	126.9±37.2^b^	<0.001
Mechanical ventilator^†^, No. (%)	78 (14.0)^a^	66 (25.0)^b^	63 (56.2)^c^	<0.001
Bacteremia, No. (%)	29 (5.2)	23 (8.7)	10 (8.9)	0.099

The values with different superscript letters in a column are significantly different (*P*<0.05).

Abbreviations: CAP, community-acquired pneumonia; HCAP, healthcare-associated pneumonia; HAP, hospital-acquired pneumonia; SD, standard deviation; BMI, body mass index; COPD, chronic obstructive pulmonary disease; HIV, human immunodeficiency virus; IV, intravenous; MRSA, methicillin-resistant *Staphylococcus aureus*; *P*. *aeruginosa*, *Pseudomonas aeruginosa*.

* Tracheostomy undergone before the diagnosis of pneumonia was investigated.

† The number included both the cases on MV after the diagnosis of pneumonia and those with VAP.

When comparing the disease severity of pneumonia, the HCAP and HAP cases were more severe than the CAP cases (mean PSI, 123.5±39.9 vs. 126.9±37.2 vs. 106.8±36.1, *P*<0.001) (Table 1). PSI was indistinguishable between the HCAP and HAP cases, but the rate of mechanical ventilation was higher in HAP than in HCAP and CAP (56.2% vs. 25.0% vs. 14.0%, *P*<0.001).

### Microbiological distribution and antibiotic resistance of causative agents

The microbiological distribution of each pneumonia group is presented in Table 2. The causative agents were identified in 46.3% of the patients (n = 432). Of the identified pathogens, 374 (86.6%) were isolated by conventional culture, and 58 (13.4%) were identified by serologic tests, urinary antigen tests, or PCR. Respiratory viral PCR was performed on 429 patients, 187 patients, and 56 patients with the positive rate of 6.3%, 2.1%, and 0% in CAP, HCAP and HAP cases, respectively. Microbiological tests for atypical pathogens were performed on 385 patients, 176 patients, and 50 patients with the positive rate of 5.2%, 2.3%, and 0% in CAP, HCAP, and HAP cases, respectively. The identification rate of causative agents was the highest in HAP (61.6%) and the lowest in CAP (40.4%) (*P*<0.001). As for the antibiotic susceptibility to the standard CAP regimen, 20.9% pathogens (195/933) were susceptible, while 20.2% (188/933) were non-susceptible. The standard CAP regimen-susceptible rate was similar among the CAP, HCAP, and HAP cases (21.2% vs. 19.7% vs. 22.3%, *P* = 0.821). However, the standard CAP regimen non-susceptibility rate was higher in the HAP and HCAP cases than in the CAP cases (38.4% vs. 28.4% vs. 12.6%, *P*<0.001). In the CAP and HCAP cases, *S*. *pneumoniae* (7.4% vs. 5.7%, *P* = 0.308) and *P*. *aeruginosa* (9.2% vs. 18.6%, *P*<0.001) were the most common Gram-positive and Gram-negative pathogens, respectively. In contrast, *Staphylococcus aureus* (MSSA 2.7%; MRSA 2.4%) and CRAB (20.5%) were the most common Gram-positive and Gram-negative pathogens in cases of HAP. Notably, the proportion of *P*. *aeruginosa* was significantly higher in the HCAP cases than in the HAP and CAP cases (18.6% vs. 8.0% vs. 9.2%, *P*<0.001), while the proportion of CRAB was remarkably higher in the HAP cases (20.5%) than in the other groups *(P<*0.001). Although generally low, the MRSA infection rate was higher in the HAP and HCAP cases than in the CAP cases (3.6% vs. 4.2% vs. 1.3%, *P* = 0.025). Among the 432 pathogen-identified cases, 188 pneumonia cases (43.5%) were non-susceptible to the standard CAP regimen: 31.1% (70/225) CAP, 54.3% (75/138) HCAP, and 62.3% (43/69) HAP cases.

**Table 2:** Microbiological distribution of hospitalized pneumonia based on the antibiogram.

No. (%)	CAP* (n = 557)	HCAP (n = 264)	HAP (n = 112)	Total (n = 933)	*p-*value
Causative agents identified	225 (40.4)^a^	138 (52.3)^b^	69 (61.6)^b^	432 (46.3)	<0.001
Standard CAP regimen^†^ susceptible bacteria	118 (21.2)	52 (19.7)	25 (22.3)	195 (20.9)	0.821
*Streptococcus pneumoniae*	41 (7.4)	15 (5.7)	3 (2.7)	59 (6.3)	0.157
MSSA	11 (2.0)^a^	10 (3.8)^ab^	7 (6.3)^b^	28 (3.0)	0.036
ESBL (-) Enterobacterales	24 (4.3)	9 (3.4)	7 (6.3)	40 (4.3)	0.461
Other Gram-positive spp.	12 (2.2)	7 (2.7)	2 (1.8)	21 (2.3)	0.849
Other Gram-negative spp.	10 (1.8)	5 (1.9)	6 (5.4)	21 (2.3)	0.061
Atypical pathogens^¶^	20 (3.6)	6 (2.3)	0 (0)	26 (2.8)	0.091
Standard CAP regimen non-susceptible bacteria	70 (12.6)^a^	75 (28.4)^b^	43 (38.4)^b^	188 (20.2)	<0.001
MRSA	7 (1.3)^a^	11 (4.2)^b^	4 (3.6)^ab^	22 (2.4)	0.025
*Pseudomonas aeruginosa*	51 (9.2)^a^	49 (18.6)^b^	9 (8.0)^a^	109(11.7)	<0.001
CRAB	5 (0.9)^a^	4 (1.5)^a^	23 (20.5)^b^	32 (3.4)	<0.001
ESBL (+) Enterobacterales	4 (0.7)	6 (2.3)	3 (2.7)	13 (1.4)	0.096
Other Gram-positive spp.	2 (0.4)^a^	5 (1.9)^b^	4 (3.6)^b^	11 (1.2)	0.007
Other Gram-negative spp.	1 (0.2)	0 (0)	0 (0)	1 (0.1)	0.071
Polymicrobial^‡^	8 (1.4)	6 (2.3)	1 (0.9)	15(1.6)	0.548
Others				34 (3.6)	
Virus	27 (4.8)^a^	4 (1.5)^b^	0 (0)^b^	31 (3.3)	0.005
Fungus	2 (0.4)	1 (0.4)	0 (0)	3 (0.3)	0.814
Causative agents unidentified	332 (59.6) ^a^	126 (47.7) ^b^	43 (38.4) ^b^	501 (53.7)	<0.001

The values with different superscript letters in a column are significantly different (*p* <0.05).

Abbreviations: CAP, community-acquired pneumonia; HCAP, healthcare-associated pneumonia; HAP, hospital-acquired pneumonia; MSSA, methicillin-susceptible *Staphylococcus aureus*; spp., species; MRSA, methicillin-resistant *Staphylococcus aureus*; CRAB, carbapenem-resistant *Acinetobacter baumannii*; ESBL, extended-spectrum beta lactamase.

† CAP standard regimen includes β-lactam plus macrolide or respiratory fluoroquinolone alone.

¶ *Mycoplasma pneumoniae*, *Chlamydophila pneumoniae*, or *Legionella pneumophilia*.

‡ If two or more organisms were identified in clinical culture, they were included as polymicrobial.

§ CAP standard regimen non-susceptible organisms include MRSA, *Pseudomonas aeruginosa*, CRAB, ESBL (+) Enterobacterales, other non-susceptible Gram-positive and Gram-negative spp.

We further analyzed four subgroups of HCAP cases based on the history of LTCF residence, prior hospitalization, and prior antibiotic use. The demographics and clinical characteristics of the HCAP subgroups are shown in S1 Table. When we compared the proportion of antibiotic-resistant pathogens, *P*. *aeruginosa* was more frequently isolated in the HCAP subgroups than in the CAP group, except for subgroup 4 (Table 3). MRSA isolation was similar between the CAP group and the HCAP subgroups. Only subgroup 3 showed a higher MRSA isolation rate than the CAP group (4.5% vs. 1.6%, *P* = 0.047). In particular, *P*. *aeruginosa* (23.7% vs. 15.6%, *P* = 0.101) and standard CAP regimen non-susceptible pathogens (35.1% vs. 20.4%, *P* = 0.068) were more common in LTCF-onset HCAP (subgroup 1 and 2) than in community-onset HCAP (subgroups 3 and 4) (S2 Table).

**Table 3:** Comparison of antibiotic-resistant pathogen distribution between CAP and HCAP subgroups.

Isolation	CAP* (n = 557)	HCAP (n = 264)			
		Subgroup 1 (n = 58)	Subgroup 2 (n = 39)	Subgroup 3 (n = 110)	Subgroup 4 (n = 57)
*Pseudomonas aeruginosa* No. (%)	53 (9.5)	15 (25.9)	10 (25.6)	18 (16.4)	9 (15.8)
*p*-value	reference	<0.001	0.002	0.033	0.134
MRSA No. (%)	8 (1.6)	2 (3.4)	2 (5.1)	5 (4.5)	2 (3.5)
*p*-value	reference	0.241	0.135	0.047	0.236

Abbreviations: CAP, community-acquired pneumonia; HCAP, healthcare-associated pneumonia; MRSA, methicillin-resistant *Staphylococcus aureus*; LTCF, long-term care facility.

Subgroup 1: LTCF-onset HCAP without a history of prior hospitalization and intravenous antibiotic use within the past 90 days. Subgroup 2: LTCF-onset HCAP with a history of prior hospitalization or intravenous antibiotic use within the past 90 days. Subgroup 3: Community-onset HCAP with a history of prior hospitalization or intravenous antibiotic use within the past 90 days. Subgroup 4: Community-onset HCAP without a history of prior hospitalization and intravenous antibiotic use within the past 90 days.

We compared the rates of *P*. *aeruginosa* infection in LTCF-onset HCAP (subgroups 1 and 2) and community-onset HCAP (subgroups 3 and 4). Cases of LTCF-onset HCAP had a higher rate of *P*. *aeruginosa* infection than those of community-onset HCAP, although the difference was not statistically significant (23.7% vs. 15.6%, *P* = 0.101).

The risk factors for *P*. *aeruginosa* infection were evaluated in CAP and HCAP patients. The univariable and multivariable analysis of risk factors for *P*. *aeruginosa* infection in CAP and HCAP was shown in Table 4. Prior *P*. *aeruginosa* isolation (OR 15.32; 95% CI 6.08–27.46) followed by tube feeding (OR 6.03; 95% CI 2.04–17.83), chemotherapy (OR 2.62; 95% CI 1.23–5.59), COPD (OR 2.39; 95% CI 1.29–4.44), and LTCF (OR 2.20; 95% CI 1.09–4.47) in multivariable analysis.

**Table 4:** Univariable and multivariable analysis of risk factors for *P*. *aeruginosa* infection in CAP and HCAP patients.

	Univariable OR (95% CI)	*p-*value	Multivariable OR (95% CI)	*p-*value
Age	1.01 (1.00–1.03)	0.158		
Sex	0.57 (0.37–0.87)	0.009	0.55 (0.34–0.90)	0.016
Chronic heart diseases	1.63 (0.80–3.34)	0.182		
Neurovascular diseases	2.10 (1.33–3.33)	0.002	1.55 (0.84–2.85)	0.161
Chronic renal diseases	1.23 (0.66–2.31)	0.516		
Diabetes	0.58 (0.35–0.95)	0.032	0.48 (0.27–0.85)	0.012
COPD	2.50 (1.52–4.11)	<0.001	2.39 (1.29–4.44)	0.006
Asthma	0.37 (0.09–1.54)	0.172		
Structural lung diseases	2.32 (1.25–4.30)	0.008	1.85 (0.89–3.86)	0.101
Liver cirrhosis	1.08 (0.32–3.72)	0.898		
Hematologic malignancy	2.45 (0.65–9.20)	0.185		
Solid cancer	1.04 (0.64–1.69)	0.874		
Rheumatologic diseases	1.56 (0.44–5.53)	0.490		
Immunosuppressant use	0.84 (0.35–2.01)	0.693		
Tracheostomy*	4.16 (1.37–12.69)	0.012	0.44 (0.09–2.10)	0.301
Tube feeding	8.91 (4.16–19.10)	<0.001	6.03 (2.04–17.83)	0.001
Prior IV antibiotic use within 90 days	1.67 (0.95–2.91)	0.074		
Long-term care facility	2.61 (1.55–4.41)	<0.001	2.20 (1.09–4.47)	0.028
Prior hospitalization within 90 days	1.52 (0.89–2.59)	0.125		
Chemotherapy	2.48 (1.28–4.81)	0.007	2.62 (1.23–5.59)	0.013
Hemodialysis	0.93 (0.32–2.69)	0.890		
Prior MRSA isolation	4.10 (1.77–9.49)	0.001	1.21 (0.40–3.68)	0.737
Prior *P*. *aeruginosa* isolation	15.32 (7.97–29.43)	<0.001	12.92 (6.08–27.46)	<0.001

Abbreviations: *P*. *aeruginosa*, *Pseudomonas aeruginosa*; CAP, community-acquired pneumonia; HCAP, healthcare-associated pneumonia; OR, odds ratio; CI, confidence interval; COPD, Chronic obstructive pulmonary disease; MRSA, methicillin-resistant *Staphylococcus aureus*.

* Tracheostomy undergone before the diagnosis of pneumonia was investigated.

As for the antibiotic susceptibility of bacterial isolates, 163 cases of CAP were culture-confirmed; 55.2% (90/163) of CAP cases were non-susceptible to ceftriaxone, and 60.7% (99/163) were non-susceptible to ampicillin-sulbactam (S3 Table). However, more than 80% of culture-confirmed CAP cases (134/163) were susceptible to levofloxacin. Culture-confirmed HCAP cases showed higher rate of non-susceptibility to ceftriaxone (71.1% [86/121] vs. 55.2% [90/163], *P* = 0.006) and ampicillin-sulbactam (73.6% [89/121] vs. 60.7% [99/163], *P* = 0.024) than that of CAP cases. Moreover, the overall levofloxacin non-susceptibility rate in culture-confirmed cases was also higher in the HCAP group than in the CAP group (32.2% [39/121] vs. 17.8% [29/163], *P* = 0.005). The rate of non-susceptibility to both levofloxacin and beta-lactams was 14.7% (24/163) in culture-confirmed CAP and 30.6% (37/121) in culture-confirmed HCAP cases. In subgroup analysis, the culture-confirmed cases of LTCF-onset HCAP (subgroup 1 and 2) showed higher non-susceptibility to levofloxacin compared to the community-onset HCAP (subgroup 3 and 4) (43.6% vs. 22.7%, P = 0.014) (S2 Table).

### Treatment regimen and clinical outcome

The initial empirical antibiotic regimens are shown in S4 Table. Standard regimens (29.8%) were more frequently used for CAP, followed by β-lactams alone (22.6%) and antipseudomonal agents alone (21.9%). Patients with HCAP received antipseudomonal antibiotics most frequently (53.1%), while only 16.7% received standard regimens. Patients with HAP received antipseudomonal antibiotics (61.6%) and antipseudomonal antibiotics plus anti-MRSA antibiotics (10.7%). Overall, the empirical anti-MRSA antibiotic usage rate was low (0.6% for CAP vs. 2.7% for HCAP vs. 13.4% for HAP).

The patients with HAP showed poorer clinical outcomes than those with CAP and HCAP (Table 5). The 30-day mortality rate was significantly higher in the HAP cases and was similar between the CAP and HCAP cases (33.9% vs. 11.0% vs. 14.8%, *P*<0.001). Pneumonia-related complications, including acute kidney injury, rhabdomyolysis, and cardiovascular events, were more common in the HAP cases than in the CAP and HCAP cases.

**Table 5:** Comparison of clinical outcomes and complications of hospitalized patients with pneumonia.

	CAP*	HCAP	HAP	*p-*value
	(n = 557)	(n = 264)	(n = 112)	
Clinical outcomes				
Duration of hospitalization, days (mean±SD)	13.0±12.0^*a*^	17.5±16.4^*b*^	27.9±27.5^*c*^	<0.001
Duration of ICU admission, days (mean±SD)	2.1±6.3^*a*^	5.2±10.6^*b*^	10.1±14.0^*c*^	<0.001
Antibiotic treatment duration, days (mean±SD)	14.1±7.7^*a*^	16.4±10.7^*b*^	19.3±13.0^*b*^	<0.001
Rehospitalization within a year, No. (%)	146 (26.2)^*a*^	98 (37.1)^*b*^	32 (28.6)^*b*^	0.006
30-day mortality, No. (%)	61 (11.0)^*a*^	39 (14.8)^*a*^	38 (33.9)^*b*^	<0.001
Complications, no. (%)				
Acute kidney injury	89 (16.0)^*a*^	60 (22.7)^*b*^	49 (43.8)^*c*^	<0.001
Rhabdomyolysis	22 (3.9)^*a*^	8 (3.0)^*a*^	15 (13.4)^*b*^	<0.001
Lung abscess	8 (1.4)	1 (0.4)	2 (1.8)	0.346
Empyema	64 (11.5)	24 (9.1)	7 (6.2)	0.194
Cardiovascular event	53 (9.5)^*a*^	30 (11.4)^*a*^	23 (20.5)^*b*^	0.004

The values with different superscript letters in a column are significantly different (*P*<0.05).

Abbreviations: CAP, community-acquired pneumonia; HCAP, healthcare-associated pneumonia; HAP, hospital-acquired pneumonia; SD, standard deviation.

## Discussion

The key findings of this study are as follows: (1) there was a different microbiological distribution among cases of CAP, HCAP, and HAP; (2) *P*. *aeruginosa* was the most common causative pathogen of CAP and HCAP, while CRAB was predominant in HAP; (3) cases of HCAP showed higher rates of *P*. *aeruginosa* and MRSA infection than the CAP cases; (4) despite high rates of non-susceptibility, ceftriaxone and ampicillin-sulbactam were commonly used as empirical treatment regimens for CAP and HCAP; (5) Prior isolation of *P*. *aeruginosa* was the most important risk factor for *P*. *aeruginosa* in CAP and HCAP cases.

As for the microbiologic distribution and antibiotic resistance, the results of this study are consistent with those of the previous studies showing higher resistance rates in HCAP and HAP compared to that in CAP [6, 7, 12–14]. In previous studies, the most common causative agents of CAP and HCAP have been reported as *S*. *pneumoniae*, and in some studies, *K*. *pneumoniae* [6, 7, 12–18]. In this study, although *S*. *pneumoniae* (7.4%) is still the most common Gram-positive pathogen in CAP, the proportion was lower than that reported in previous studies (7.9–25.0%) [6, 7, 12–15, 17]. In a recent prospective cohort study in South Korea between September 2015 and August 2017, pneumococcal CAP accounted for 9.4% of the total 2,669 cases of CAP [19]. In that study, the estimated incidence of pneumococcal CAP was lower than that in the previous reports, probably because of the introduction of the National Immunization Program for pneumococcal diseases, which included 10-valent pneumococcal conjugate vaccine (PCV10)/13-valent pneumococcal conjugate vaccine 13 (PCV13) for children and 23-valent pneumococcal polysaccharide vaccine (PPSV23) for the elderly. As reported previously, an indirect protective effect for pneumococcal infection can be seen in adults when the pediatric PCV immunization rate reaches over 60%, and the indirect herd effect increases to a maximal level over a 7-year period [20]. In South Korea, pediatric PCV 10/PCV 13 uptake rates reached over 60% in 2012; therefore, the indirect protective effects in adults would have maximized in 2019. Thus, high uptake of the pneumococcal vaccine in South Korea may be, at least in part, responsible for the declining prevalence of this pathogen in CAP. However, serotype replacement is proceeding under vaccine pressure; therefore, the epidemiology of CAP can further evolve over time.

In previous studies, *P*. *aeruginosa* has been reported as a causative agent in 0.8–4.0% of CAP and 3.0–8.9% of HCAP cases [6, 7, 12–18]. Interestingly, in this study, *P*. *aeruginosa* was the most common pathogen in the CAP (9.2%) and HCAP (18.6%) cases. This result differs from that of the previous studies. The study setting and severity of pneumonia might have affected the difference in results. In a recently published study used the Premier Healthcare Database drawn from the 177 hospitals in Unite states, *P*. *aeruginosa* was the most common pathogen recovered from respiratory culture in non-severe CAP (4.9%) and severe CAP (6.1%). In addition, patients with severe CAP were about three times more likely to have antibiotic-resistant infection compared with those with non-severe CAP [21]. In our study, study subjects were patients with pneumonia with high comorbidities who were hospitalized in a tertiary hospital. In addition, the patients in this study had higher PSI (mean PSI 106±36.1) than those in the other studies (82.0–97.4) [6, 7, 12–15]. The introduction of pneumococcal vaccination might have reduced the rate of *S*. *pneumoniae* infection, allowing the causative agents to be redistributed with increasing *P*. *aeruginosa* infection. However, as this is a retrospective single-center study, further prospective multi-center studies are necessary. *P*. *aeruginosa* was more frequently isolated in the HCAP cases than in the CAP cases (*P*<0.001). Prior isolation of *P*. *aeruginosa* in respiratory sample was the most important risk factor for *P*. *aeruginosa* infection. However, the rate of prior isolation of *P*. *aeruginosa* was not different between CAP and HCAP cases. Other risk factors of COPD, LTCF or chemotherapy might affect the higher rate of *P*. *aeruginosa* infection in CAP and HCAP cases, requiring further validation. Considering the higher rate of *P*. *aeruginosa* infection in HCAP cases, antipseudomonal antibiotics might be considered as empirical agents.

In contrast, the MRSA infection rate was quite low in both CAP (1.3%) and HCAP (4.2%) cases, contrary to the expectations. In the previous studies, the proportion of MRSA infection was 0–2.6% in CAP cases and 1.3–8.0% in HCAP cases [6, 7, 12–14, 16, 18]. Given that MRSA infection is rare in CAP and HCAP cases, empirical anti-MRSA treatment should be considered only in patients at a high risk for MRSA infection, including those with prior respiratory isolation of MRSA within the past year, severe CAP with recent hospitalization and intravenous antibiotics within the past 90 days, and locally validated risk factors for MRSA [10].

CRAB is one of the most common pathogen of HAP. CRAB has been a problematic microorganism in nosocomial pneumonia of South Korea as reported in a multi-center (13 tertiary or university hospitals) cohort study. In that study, *A*. *baumannii* accounted for 31.8% (68/211) of identified pathogens in nosocomial pneumonia, and 98.5% (67/68) of *A*. *baumannii* were multi-drug resistant [22]. CRAB is mainly transmitted through direct or indirect contact with a contaminated environment. Because CRAB is resistant to several antibiotics and disinfectants, they are prone to colonization in healthcare facilities, especially in ICUs [23]. Most hospitals in South Korea operate multi-patient rooms, which make it difficult to prevent the transmission of CRAB [24].

All cases of viral and atypical infections were observed only in CAP and HCAP, with the higher frequency in CAP than in HCAP. For HAP cases, the tests were performed in about half of all cases, but all of them were negative. The proportion of viral and atypical infections in our study was lower than those in other studies. In the previous studies, however, respiratory syncytial virus (RSV), parainfluenza virus, and adenovirus were reported to cause viral pneumonia in the immunocompromised inpatients [25–27]. The proportion of viral and atypical pneumonia might be variable depending on the patient population of each hospital.

In this study, 40.1% of bacteria were isolated from conventional cultures. In the susceptibility test for bacterial isolates, the non-susceptibility rates to ceftriaxone (71.1% vs. 55.2%, *P* = 0.006) and ampicillin-sulbactam (73.6% vs. 60.7%, *P* = 0.024) were significantly higher in HCAP cases than in CAP cases. In addition, the non-susceptibility rate to levofloxacin was 17.8% in CAP and 32.3% in HCAP cases (*P* = 0.005). The susceptibility rate to levofloxacin was relatively higher than that to β-lactam antibiotics. Although antibiotic susceptibility was compared only for identified isolates, the results suggest that there is a significant difference in antibiogram between CAP and HCAP.

HCAP is a highly heterogeneous disease that is similar to CAP in some aspects and close to HAP in others. Thus, we further categorized HCAP into four subgroups according to the history of LTCF residence, prior hospitalization, and prior antibiotic use. The rate of non-susceptibility to levofloxacin was higher in LTCF-onset HCAP than in community-onset HCAP. In a previous study, antibiotic resistance rates were remarkably higher in LTCF residents than in hospitalized patients and outpatients [28, 29]. Moreover, antibiotics are frequently prescribed in LTCFs, especially for urinary tract infections, aspiration pneumonia, and sore infections [30, 31]. The colonization of antibiotic-resistant bacteria and frequent exposure to antibiotic agents have the potential to increase the risk of antibiotic-resistant bacterial infections. This suggests that physicians should consider LTCF residence when choosing an empirical antibiotic regimen. In our study, LTCF residence was an independent risk factor for *P*. *aeruginosa* infection.

This study has some limitations. First, as the pneumonia cases were identified by the investigators retrospectively, selection bias may exist. To reduce this, two experienced infectious disease doctors reviewed all the cases and resolved disagreements through discussion. Second, this was a single-center study. Therefore, the results of this study should be interpreted carefully and further prospective multi-center studies are needed. Third, the microbial identification rate was 46.3% and the remaining 53.7% of causative agents were unknown. Molecular diagnostic tests and advanced urinary antigen detection tests would be useful to increase the microbiological identification rate in future studies. Fourth, we classified *P*. *aeruginosa* as a non-susceptible organism to standard CAP regimen regardless of the in vitro susceptibility to fluoroquinolone. *P*. *aeruginosa* is non-susceptible to ceftriaxone plus macrolide, and fluoroquinolone monotherapy is not recommended as an empiric anti-pseudomonal agent according to the 2019 ATS/IDSA guidelines for CAP [10]. Nevertheless, some fluoroquinolone-susceptible *P*. *aeruginosa* CAP cases might be misclassified as non-susceptible infection. Finally, we did not analyze HAP cases in detail, classifying into subgroups. There would be much difference in the distribution of causative agents, risk factors, and prognosis between mild and severe HAP cases requiring mechanical ventilation.

In conclusion, HCAP cases showed a higher rate (28.4%) of infection by antibiotic-resistant pathogens. In particular, *P*. *aeruginosa* was the most common pathogen in CAP (7.4%) and HCAP (18.6%). The proportion of non-susceptibility to β-lactam and levofloxacin was higher in HCAP compared to CAP. The rate of non-susceptibility to levofloxacin was higher in LTCF-onset HCAP than in community-onset HCAP. Either piperacillin-tazobactam or cefepime should be considered in the treatment of LTCF-onset HCAP based on the local antibiogram.

## Supporting information

S1 Table Demographic and clinical characteristics of patients with HCAP by risk category. (DOCX)

S2 Table Comparison of antibiotic resistance and clinical outcomes between long-term care facility onset healthcare-associated pneumonia and community-onset healthcare-associated pneumonia. (DOCX)

S3 Table Comparison of standard antibiotic susceptibility for isolated bacterial pathogens^†^ between CAP and HCAP subgroups. (DOCX)

S4 Table Comparison of antibiotic treatment regimens for pneumonia. (DOCX)

## References

[1] WHO. Global Health Estimates 2020: Deaths by Cause, Age, Sex, by Country and by Region, 2000–2019 Geneva, World Health Organization 2020 [Available from: https://www.who.int/data/gho/data/themes/mortality-and-global-health-estimates/ghe-leading-causes-of-death].

[2] American Thoracic S, Infectious Diseases Society of A. Guidelines for the management of adults with hospital-acquired, ventilator-associated, and healthcare-associated pneumonia. Am J Respir Crit Care Med. 2005;171(4):388–416. doi: 10.1164/rccm.200405-644ST 15699079

[3] Chalmers JD, Rother C, Salih W, Ewig S. Healthcare-associated pneumonia does not accurately identify potentially resistant pathogens: a systematic review and meta-analysis. Clin Infect Dis. 2014;58(3):330–9. doi: 10.1093/cid/cit734 24270053

[4] Kalil AC, Metersky ML, Klompas M, Muscedere J, Sweeney DA, Palmer LB, et al. Management of Adults With Hospital-acquired and Ventilator-associated Pneumonia: 2016 Clinical Practice Guidelines by the Infectious Diseases Society of America and the American Thoracic Society. Clin Infect Dis. 2016;63(5):e61–e111. doi: 10.1093/cid/ciw353 27418577 PMC4981759

[5] Nseir S, Grailles G, Soury-Lavergne A, Minacori F, Alves I, Durocher A. Accuracy of American Thoracic Society/Infectious Diseases Society of America criteria in predicting infection or colonization with multidrug-resistant bacteria at intensive-care unit admission. Clin Microbiol Infect. 2010;16(7):902–8. doi: 10.1111/j.1469-0691.2009.03027.x 19694760

[6] Ahn JH, Lee KH, Chung JH, Shin KC, Lee CK, Kim HJ, et al. Clinical characteristics and prognostic risk factors of healthcare-associated pneumonia in a Korean tertiary teaching hospital. Medicine (Baltimore). 2017;96(42):e8243. doi: 10.1097/MD.0000000000008243 29049213 PMC5662379

[7] Kim ES, Park KU, Lee SH, Lee YJ, Park JS, Cho YJ, et al. Comparison of viral infection in healthcare-associated pneumonia (HCAP) and community-acquired pneumonia (CAP). PLoS One. 2018;13(2):e0192893. doi: 10.1371/journal.pone.0192893 29447204 PMC5813982

[8] Lopes M, Alves Silva G, Nogueira RF, Marado D, Goncalves J, Athayde C, et al. Incidence of Antibiotic Treatment Failure in Patients with Nursing Home-Acquired Pneumonia and Community Acquired Pneumonia. Infect Dis Rep. 2021;13(1):33–44. doi: 10.3390/idr13010006 33466353 PMC7838805

[9] Clinical and Laboratory Standards Institute. Performance standards for antimicrobial susceptibility testing. Wayne, PA: Clinical Laboratory Standards Institute; 2017. p. M100–S27. 10.1128/JCM.00213-21 PMC8601225 34550809

[10] Metlay JP, Waterer GW, Long AC, Anzueto A, Brozek J, Crothers K, et al. Diagnosis and Treatment of Adults with Community-acquired Pneumonia. An Official Clinical Practice Guideline of the American Thoracic Society and Infectious Diseases Society of America. Am J Respir Crit Care Med. 2019;200(7):e45–e67. doi: 10.1164/rccm.201908-1581ST 31573350 PMC6812437

[11] Khwaja A. KDIGO clinical practice guidelines for acute kidney injury. Nephron Clin Pract. 2012;120(4):c179–84. doi: 10.1159/000339789 22890468

[12] Jung JY, Park MS, Kim YS, Park BH, Kim SK, Chang J, et al. Healthcare-associated pneumonia among hospitalized patients in a Korean tertiary hospital. BMC Infect Dis. 2011;11:61. doi: 10.1186/1471-2334-11-61 21396096 PMC3063837

[13] Maruyama T, Fujisawa T, Okuno M, Toyoshima H, Tsutsui K, Maeda H, et al. A new strategy for healthcare-associated pneumonia: a 2-year prospective multicenter cohort study using risk factors for multidrug-resistant pathogens to select initial empiric therapy. Clin Infect Dis. 2013;57(10):1373–83. doi: 10.1093/cid/cit571 23999080

[14] Park HK, Song JU, Um SW, Koh WJ, Suh GY, Chung MP, et al. Clinical characteristics of health care-associated pneumonia in a Korean teaching hospital. Respir Med. 2010;104(11):1729–35. doi: 10.1016/j.rmed.2010.06.009 20605087

[15] Fukuyama H, Yamashiro S, Kinjo K, Tamaki H, Kishaba T. Validation of sputum Gram stain for treatment of community-acquired pneumonia and healthcare-associated pneumonia: a prospective observational study. BMC Infect Dis. 2014;14:534. doi: 10.1186/1471-2334-14-534 25326650 PMC4287475

[16] Ito A, Ishida T, Tachibana H, Nakanishi Y, Yamazaki A, Washio Y. Is antipseudomonal antibiotic treatment needed for all nursing and healthcare-associated pneumonia patients at risk for antimicrobial resistance? J Glob Antimicrob Resist. 2020;22:441–7. doi: 10.1016/j.jgar.2020.04.021 32339851

[17] Kamata K, Suzuki H, Kanemoto K, Tokuda Y, Shiotani S, Hirose Y, et al. Clinical evaluation of the need for carbapenems to treat community-acquired and healthcare-associated pneumonia. J Infect Chemother. 2015;21(8):596–603. doi: 10.1016/j.jiac.2015.05.002 26070781

[18] Yamagata A, Ito A, Nakanishi Y, Ishida T. Prognostic factors in nursing and healthcare-associated pneumonia. J Infect Chemother. 2020;26(6):563–9. doi: 10.1016/j.jiac.2020.01.009 32067902

[19] Heo JY, Seo YB, Jeong HW, Choi MJ, Min KH, Choi WS, et al. Epidemiology of community-acquired pneumonia in the era of extended serotype-covering multivalent pneumococcal conjugate vaccines. Vaccine. 2020;38(49):7747–55. doi: 10.1016/j.vaccine.2020.10.046 33164798

[20] Pilishvili T, Lexau C, Farley MM, Hadler J, Harrison LH, Bennett NM, et al. Sustained reductions in invasive pneumococcal disease in the era of conjugate vaccine. J Infect Dis. 2010;201(1):32–41. doi: 10.1086/648593 19947881

[21] Haessler S, Guo N, Deshpande A, Zilberberg MD, Lagu T, Lindenauer PK, et al. Etiology, Treatments, and Outcomes of Patients With Severe Community-Acquired Pneumonia in a Large U.S. Sample. Crit Care Med. 2022. 10.1097/CCM.0000000000005498 PMC9233133 35191410

[22] Ko RE, Min KH, Hong SB, Baek AR, Lee HK, Cho WH, et al. Characteristics, Management, and Clinical Outcomes of Patients with Hospital-Acquired and Ventilator-Associated Pneumonia: A Multicenter Cohort Study in Korea. Tuberc Respir Dis (Seoul). 2021;84(4):317–25. doi: 10.4046/trd.2021.0018 34134465 PMC8497766

[23] Peleg AY, Seifert H, Paterson DL. Acinetobacter baumannii: emergence of a successful pathogen. Clin Microbiol Rev. 2008;21(3):538–82. doi: 10.1128/CMR.00058-07 18625687 PMC2493088

[24] Choi JY, Kwak YG, Yoo H, Lee SO, Kim HB, Han SH, et al. Trends in the distribution and antimicrobial susceptibility of causative pathogens of device-associated infection in Korean intensive care units from 2006 to 2013: results from the Korean Nosocomial Infections Surveillance System (KONIS). J Hosp Infect. 2016;92(4):363–71. doi: 10.1016/j.jhin.2015.12.012 26876746

[25] Ison MG, Hirsch HH. Community-Acquired Respiratory Viruses in Transplant Patients: Diversity, Impact, Unmet Clinical Needs. Clin Microbiol Rev. 2019;32(4). doi: 10.1128/CMR.00042-19 31511250 PMC7399564

[26] Jensen TO, Stelzer-Braid S, Willenborg C, Cheung C, Andresen D, Rawlinson W, et al. Outbreak of respiratory syncytial virus (RSV) infection in immunocompromised adults on a hematology ward. J Med Virol. 2016;88(10):1827–31. doi: 10.1002/jmv.24521 26990584

[27] Shah DP, Shah PK, Azzi JM, Chemaly RF. Parainfluenza virus infections in hematopoietic cell transplant recipients and hematologic malignancy patients: A systematic review. Cancer Lett. 2016;370(2):358–64. doi: 10.1016/j.canlet.2015.11.014 26582658 PMC4684719

[28] Aschbacher R, Pagani L, Migliavacca R, Pagani L, group GLw. Recommendations for the surveillance of multidrug-resistant bacteria in Italian long-term care facilities by the GLISTer working group of the Italian Association of Clinical Microbiologists (AMCLI). Antimicrob Resist Infect Control. 2020;9(1):106. doi: 10.1186/s13756-020-00771-0 32660605 PMC7356128

[29] McKinnell JA, Singh RD, Miller LG, Kleinman K, Gussin G, He J, et al. The SHIELD Orange County Project: Multidrug-resistant Organism Prevalence in 21 Nursing Homes and Long-term Acute Care Facilities in Southern California. Clin Infect Dis. 2019;69(9):1566–73. doi: 10.1093/cid/ciz119 30753383 PMC7320073

[30] Cohen CC, Dick AW, Agarwal M, Gracner T, Mitchell S, Stone PW. Trends in antibiotics use among long-term US nursing-home residents. Infect Control Hosp Epidemiol. 2021;42(3):311–7. doi: 10.1017/ice.2020.422 32935657 PMC7960578

[31] Thompson ND, Stone ND, Brown CJ, Penna AR, Eure TR, Bamberg WM, et al. Antimicrobial Use in a Cohort of US Nursing Homes, 2017. JAMA. 2021;325(13):1286–95. doi: 10.1001/jama.2021.2900 33821897 PMC8025112

