# Supplementary material for: Risk factor-based analysis of community-acquired pneumonia, healthcare-associated pneumonia and hospital-acquired pneumonia: Microbiological distribution, antibiotic resistance, and clinical outcomes
Source: PLoS One. 2022 Jun 29;17(6):e0270261. doi: 10.1371/journal.pone.0270261 (PMC9242491; doi:10.1371/journal.pone.0270261)
Supplement: S1 Table — (DOCX) [file pone.0270261.s001.docx]

**S1 Table. Demographic and clinical characteristics of patients with HCAP by risk category.**

|  | | HCAP (n=264) | | | | |
| --- | --- | --- | --- | --- | --- | --- |
|  | Subgroup 1 (n=58) | | Subgroup 2 (n=39) | Subgroup 3 (n=110) | Subgroup 4 (n=57) | *p-v*alue |
| Demographics |  | |  |  |  |  |
| Male sex, No. (%) | 35 (60.9) | | 23 (59.0) | 62 (56.4) | 37 (64.9) | 0.762 |
| Age, year (mean ± SD) | 78.6 ± 10.2^a^ | | 75.1 ± 11.1^ab^ | 69 ± 13.8^bc^ | 67.4 ± 11.0^c^ | 0.017 |
| BMI | 19.7 ± 3.9^a^ | | 20.1 ± 2.7^ab^ | 22.9 ± 4.1^b^ | 22.5 ± 4.9^b^ | 0.011 |
| Medical comorbidities, No. (%) |  | |  |  |  |  |
| Chronic heart diseases | 6 (10.3) | | 6 (15.4) | 8 (7.3) | 6 (10.5) | 0.529 |
| Neurovascular diseases | 40 (69.0)^a^ | | 21 (53.8)^a^ | 17 (15.5)^b^ | 5 (8.8)^b^ | <0.001 |
| Chronic renal diseases | 4 (6.9)^a^ | | 6 (15.4)^a^ | 11 (10.0)^a^ | 29 (50.9)^b^ | <0.001 |
| Diabetes | 17 (29.3) | | 17 (43.6) | 35 (31.8) | 23 (40.4) | 0.348 |
| COPD | 3(5.2) | | 3 (7.7) | 15 (13.6) | 9 (15.8) | 0.221 |
| Asthma | 2 (3.4) | | 2 (5.1) | 9 (8.2) | 1 (1.8) | 0.303 |
| Structural lung diseases | 2 (3.4) | | 2 (5.1) | 7 (6.4) | 2 (3.5) | 0.800 |
| Liver cirrhosis | 0 (0.0) | | 0 (0.0) | 4 (3.6) | 2 (3.5) | 0.312 |
| Hematologic malignancy | 2 (3.4) | | 0 (0.0) | 2 (1.8) | 1 (1.8) | 0.678 |
| Solid cancer | 9 (15.5)^a^ | | 6 (15.4)^a^ | 41 (37.3)^b^ | 35 (61.4^)c^ | <0.001 |
| Rheumatologic diseases | 1 (1.7) | | 0 (0.0) | 3 (2.7) | 0 (0.0) | 0.462 |
| Immunosuppressant use | 1 (1.7) | | 1 (2.6) | 9 (8.2) | 5 (8.8) | 0.225 |
| Solid organ transplantation | 0 (0.0) | | 1 (2.5) | 0 (0.0) | 0 (0.0) | 0.122 |
| Charlson Comorbidity Index | 5.1 ± 1.9 | | 5.4 ± 2.5 | 5.0 ± 3.2 | 6.1 ± 1.9 | 0.057 |
| Medical device use, No. (%) |  | |  |  |  |  |
| Tracheostomy^*^ | 3 (5.2)^ab^ | | 6 (15.4)^b^ | 2 (1.8)^a^ | 0 (0.0)^a^ | 0.001 |
| Tube feeding | 9 (15.5)^a^ | | 10 (25.6)^a^ | 5 (4.5)^b^ | 0 (0.0)^b^ | <0.001 |
| Antibiotic resistance related risk factor, No. (%) | | |  |  |  |  |
| Prior IV antibiotic use within 90 days | 0 (0)^a^ | | 27 (69.2)^c^ | 75 (68.2)^b^ | 0 (0)^a^ | <0.001 |
| Long-term care facility | 58 (100)^a^ | | 39 (100)^a^ | 0 (0)^b^ | 0 (0)^b^ | <0.001 |
| Prior hospitalization within 90 days | 0 (100)^a^ | | 30 (76.9)^b^ | 92 (83.6)^b^ | 0 (0)^c^ | <0.001 |
| Chemotherapy | 0 (0)^a^ | | 2 (5.1)^a^ | 22 (20)^b^ | 30 (52.6)^c^ | <0.001 |
| Hemodialysis | 2 (3.4)^a^ | | 2 (5.1)^a^ | 4 (3.6)^a^ | 27 (47.4)^b^ | <0.001 |
| Prior MRSA isolation | 3 (5.4) | | 8 (7.3) | 3 (7.7) | 0 (0) | 0.213 |
| Prior *P. aeruginosa* isolation | 2 (3.6) | | 10 (9.5) | 4 (10.3) | 2 (3.6) | 0.308 |
| Pneumonia Severity |  | |  |  |  |  |
| CURB65, median (IQR) | 2 (2-3)^a^ | | 2 (1-3)^ab^ | 2 (1-2)^b^ | 2 (1-2)^b^ | <0.001 |
| Pneumonia severity index, mean±SD | 135.5 ± 34.1^a^ | | 131.0 ± 35.6^ab^ | 115.4 ± 45.9^b^ | 121.7 ± 32.0^ab^ | 0.006 |
| Mechanical ventilator†, No. (%) | 21 (36.2)^a^ | | 14 (35.9)^a^ | 19 (17.3)^b^ | 12 (21.1)^ab^ | 0.016 |
| Bacteremia, No. (%) | 6 (10.3) | | 4 (10.3) | 8 (7.3) | 5 (8.8) | 0.897 |
| Clinical outcomes |  | |  |  |  |  |
| Duration of ICU admission,  Days (mean±SD) | 9.3 ± 13.7 | | 7.6 ± 11.9 | 3.3 ± 8.2 | 3.1 ± 8.9 | 0.285 |
| 30-day mortality, No. (%) | 7 (12.1) | | 5 (12.8) | 17 (15.5) | 10 (17.5) | 0.839 |
| The values with different superscript letters in a column are significantly different (*P*<0.05)  Abbreviations: HCAP, healthcare-associated pneumonia; SD, standard deviation; BMI, body mass index; COPD, chronic obstructive pulmonary diseases; HIV, human immunodeficiency virus; IV, intravenous; ICU, intensive care unit; LTCF, long-term care facility; MRSA, methicillin-resistant *Staphylococcus aureus; P. aeruginosa, Pseudomonas aeruginosa*.  Subgroup 1: LTCF-onset HCAP without a history of prior hospitalization and intravenous antibiotic use within the past 90 days. Subgroup 2: LTCF-onset HCAP with a history of prior hospitalization or intravenous antibiotic use within the past 90 days. Subgroup 3: Community-onset HCAP with a history of prior hospitalization or intravenous antibiotic use within the past 90 days. Subgroup 4: Community-onset HCAP without a history of prior hospitalization and intravenous antibiotic use within the past 90 days.  * Tracheostomy undergone before the diagnosis of pneumonia was investigated  † The number included both the cases on MV after the diagnosis of pneumonia and those with VAP | | | | | | |
