# Supplementary material for: Risk factor-based analysis of community-acquired pneumonia, healthcare-associated pneumonia and hospital-acquired pneumonia: Microbiological distribution, antibiotic resistance, and clinical outcomes
Source: PLoS One. 2022 Jun 29;17(6):e0270261. doi: 10.1371/journal.pone.0270261 (PMC9242491; doi:10.1371/journal.pone.0270261)
Supplement: S2 Table — (DOCX) [file pone.0270261.s002.docx]

**S2 Table. Comparison of antibiotic resistance and clinical outcomes between long-term care facility onset healthcare-associated pneumonia and community-onset healthcare-associated pneumonia.**

|  | LTCF-onset HCAP (n=97) | Community-onset HCAP (n=167) | | *p-v*alue |
| --- | --- | --- | --- | --- |
| Antibiotic resistance related risk factor, No. (%) |  |  |  | |
| Prior IV antibiotic use within 90 days | 27 (27.8) | 75 (44.0) | 0.006 | |
| Long-term care facility | 97 (100) | 0 (0) | <0.001 | |
| Prior hospitalization within 90 days | 30 (30.9) | 92 (55.1) | <0.001 | |
| Chemotherapy | 2 (2.1) | 52 (31.1) | <0.001 | |
| Hemodialysis | 4 (4.1) | 31 (18.6) | 0.001 | |
| Prior MRSA isolation | 6 (6.2) | 8 (4.8) | 0.626 | |
| Prior *P. aeruginosa* isolation | 6 (6.2) | 12 (7.2) | 0.756 | |
| Pneumonia severity |  |  |  | |
| CURB65, median (IQR) | 2 (1-3) | 2 (1-3) | 0.360 | |
| Pneumonia severity index, mean ± SD | 133.7±34.6 | 117±41.6 | 0.053 | |
| Mechanical ventilator^*^, No. (%) | 35 (36.1) | 31 (18.6) | 0.002 | |
| Bacteremia, No. (%) | 13 (10.3) | 13 (7.8) | 0.483 | |
| Charlson comorbidity index | 5.2±2.1 | 5.4±2.9 | 0.002 | |
| Causative agents  Standard CAP regimen-susceptible bacteria | 18 (18.6) | 34 (20.4) | 0.723 | |
| *Streptococcus pneumoniae* | 5 (5.2) | 10 (6.0) | 0.778 | |
| MSSA | 6 (6.2) | 4 (2.4) | 0.179 | |
| ESBL (-) Enterobacterales | 3 (3.1) | 6 (3,8) | 1.000 | |
| Others | 4 (4.1) | 8 (4.8) | 1.000 | |
| Atypical | 0 (0) | 6 (3.6) | 0.088 | |
| Standard CAP regimen non-susceptible bacteria | 34 (35.1) | 41 (24.6) | 0.068 | |
| MRSA | 4 (4.1) | 7 (4.2) | 1.000 | |
| *Pseudomonas aeruginosa*. | 23 (23.7) | 26 (15.6) | 0.101 | |
| CRAB | 2 (2.1) | 2 (1.2) | 0.626 | |
| ESBL (+) Enterobacterales | 4 (4.1) | 2 (1.2) | 0.197 | |
| Others | 1 (1.0) | 4 (2.4) | 0.655 | |
| Antibiotic non-susceptibility |  |  |  | |
| Ceftriaxone, No. (%) | 42 (76.4) | 44 (66.7) | 0.541 | |
| Levofloxacin, No. (%) | 24 (43.6) | 15 (22.7) | 0.014 | |
| Ampicillin-sulbactam, No. (%) | 43 (78.2) | 46 (69.7) | 0.292 | |
| Complications, no. (%) |  |  |  | |
| Acute kidney injury | 33 (34.0) | 27 (16.2) | 0.001 | |
| Rhabdomyolysis | 2 (2.1) | 6 (3.6) | 0.484 | |
| Lung abscess | 0 (0) | 1 (0.6) | 0.445 | |
| Empyema | 11 (11.3%) | 13 (7.8) | 0.333 | |
| Cardiovascular events | 11 (11.3) | 19 (11.4) | 0.993 | |
| Clinical outcomes |  |  |  | |
| Duration of hospitalization, days (mean ± SD) | 17.5±10.4 | 15.8±10.8 | 0.470 | |
| Duration of ICU admission, days (mean ± SD) | 8.6±13.0 | 3.2±8.3 | <0.001 | |
| 30-day mortality | 12 (12.4) | 27 (16.2) | 0.402 | |
| Rehospitalization within a year | 30 (35.3) | 67 (47.9) | 0.065 | |
| Abbreviations: LTCF, long-term care facility; HCAP, healthcare-associated pneumonia; SD, standard deviation; IV, intravenous; MRSA, methicillin-resistant *Staphylococcus aureus; P. aeruginosa, Pseudomonas aeruginosa*; MSSA, methicillin-susceptible *Staphylococcus aureus*; ESBL, extended-spectrum beta lactamase; CRAB, carbapenem-resistant *Acinetobactor baumamnii*; ICU, intensive care unit.  * The number included both the cases on MV after the diagnosis of pneumonia and those with VAP | | | | |
