# Supplementary material for: Risk factor-based analysis of community-acquired pneumonia, healthcare-associated pneumonia and hospital-acquired pneumonia: Microbiological distribution, antibiotic resistance, and clinical outcomes
Source: PLoS One. 2022 Jun 29;17(6):e0270261. doi: 10.1371/journal.pone.0270261 (PMC9242491; doi:10.1371/journal.pone.0270261)
Supplement: S3 Table — (DOCX) [file pone.0270261.s003.docx]

**S3 Table. Comparison of standard antibiotic susceptibility for isolated bacterial pathogens^†^ between CAP and HCAP subgroups.**

| Non-susceptible rates for antibiotics | CAP* (n=163) | HCAP | | | | |
| --- | --- | --- | --- | --- | --- | --- |
|  |  | Total (n=121) | Subgroup 1 (n=33) | Subgroup 2 (n=22) | Subgroup 3 (n=43) | Subgroup 4 (n=23) |
| Ceftriaxone, No. (%) | 90 (55.2) | 86 (71.1) | 27 (81.8) | 15 (68.2) | 30 (69.8) | 14 (60.9) |
| *p-value* | reference | 0.006 | 0.004 | 0.249 | 0.085 | 0.609 |
| Levofloxacin, No. (%) | 29 (17.8) | 39 (32.2) | 12 (36.4) | 12 (54.5) | 11 (25.6) | 4 (17.4) |
| *p-value* | reference | 0.005 | 0.017 | <0.001 | 0.251 | 1.000 |
| Ampicillin-sulbactam, No. (%) | 99 (60.7) | 89 (73.6) | 28 (84.8) | 15 (68.2) | 31 (72.1) | 15 (65.2) |
| *p*-value | reference | 0.024 | 0.008 | 0.500 | 0.170 | 0.680 |
| Abbreviations: CAP, community-acquired pneumonia; HCAP, healthcare-associated pneumonia  † Culture-confirmed cases were included.  Subgroup 1: LTCF-onset HCAP without a history of prior hospitalization and intravenous antibiotic use within the past 90 days. Subgroup 2: LTCF-onset HCAP with a history of prior hospitalization or intravenous antibiotic use within the past 90 days. Subgroup 3: Community-onset HCAP with a history of prior hospitalization or intravenous antibiotic use within the past 90 days. Subgroup 4: Community-onset HCAP without a history of prior hospitalization and intravenous antibiotic use within the past 90 days. | | | | | | |
