# Supplementary material for: Risk factor-based analysis of community-acquired pneumonia, healthcare-associated pneumonia and hospital-acquired pneumonia: Microbiological distribution, antibiotic resistance, and clinical outcomes
Source: PLoS One. 2022 Jun 29;17(6):e0270261. doi: 10.1371/journal.pone.0270261 (PMC9242491; doi:10.1371/journal.pone.0270261)
Supplement: S4 Table — (DOCX) [file pone.0270261.s004.docx]

**S4 Table. Comparison of antibiotic treatment regimens for pneumonia.**

| No. (%) | CAP (n=557) | HCAP (n=264) | HAP (n=112) | Total (n=933) | *p-v*alue |
| --- | --- | --- | --- | --- | --- |
| Treatment regimen |  |  |  |  | <0.001 |
| Standard regimen of CAP^†^ | 166 (29.8) | 44 (16.7) | 5 (4.5) | 215 (23.0) |  |
| β-lactam alone | 126 (22.6) | 35 (13.3) | 24 (21.4) | 185 (19.8) |  |
| β-lactam + FQ | 107 (19.2) | 38 (14.4) | 4 (3.6) | 149 (16.0) |  |
| β-lactam + Anti-anaerobic^‡^ | 23 (4.1) | 4 (1.5) | 4 (3.6) | 31 (3.3) |  |
| Antipseudomonal^§^ alone | 122 (21.9) | 134 (50.8) | 57 (50.9) | 313 (33.5) |  |
| Anti-MRSA^¶^ alone | 2 (0.4) | 1 (0.4) | 3 (2.7) | 6 (0.6) |  |
| Antipseudomonal + Anti-MRSA | 1 (0.2) | 6 (2.3) | 12 (10.7) | 19 (2.0) |  |
| Others | 10 (1.8) | 2 (0.8) | 3 (2.7) | 15 (1.6) |  |
| Abbreviations: CAP, community-acquired pneumonia; HCAP, healthcare-associated pneumonia; HAP, hospital-acquired pneumonia; FQ, fluoroquinolone; MRSA, methicillin-resistant *Staphylococcus aureus*.  † Standard regimen includes β-lactam plus macrolide or respiratory fluoroquinolone alone.  ‡ Clindamycin or metronidazole  § Antipseudomonal agents include ceftazidime, 4^th^ generation cephalosporin, piperacillin-tazobactam, and carbapenems (except for ertapenem).  ¶ Vancomycin, teicoplanin or linezolid | | | | | |
